# Automated signal intensity analysis of the spinal cord for detection of degenerative cervical myelopathy — a matched-pair MRI study

**DOI:** 10.1007/s00234-023-03187-w

**Published:** 2023-06-30

**Authors:** Marc Hohenhaus, Jan-Helge Klingler, Christoph Scholz, Florian Volz, Ulrich Hubbe, Jürgen Beck, Marco Reisert, Urs Würtemberger, Nico Kremers, Katharina Wolf

**Affiliations:** 1 Department of Neurosurgery, Medical Center – University of Freiburg, Faculty of Medicine, University of Freiburg, Freiburg, Germany; 2 Department of Radiology, Medical Physics, Medical Center – University of Freiburg, Faculty of Medicine, University of Freiburg, Freiburg, Germany; 3 Department of Neuroradiology, Medical Center – University of Freiburg, Faculty of Medicine, University of Freiburg, Freiburg, Germany; 4 Department of Neurology, Medical Center – University of Freiburg, Faculty of Medicine, University of Freiburg, Freiburg, Germany

**Keywords:** Degenerative cervical myelopathy, T2 hyperintensity, Myelopathy sign, Magnetic resonance imaging, Spinal cord segmentation

## Abstract

**Purpose:**

Detection of T2 hyperintensities in suspected degenerative cervical myelopathy (DCM) is done subjectively in clinical practice. To gain objective quantification for dedicated treatment, signal intensity analysis of the spinal cord is purposeful. We investigated fully automated quantification of the T2 signal intensity (T2-SI) of the spinal cord using a high-resolution MRI segmentation.

**Methods:**

Matched-pair analysis of prospective acquired cervical 3D T2-weighted sequences of 114 symptomatic patients and 88 healthy volunteers. Cervical spinal cord was segmented automatically through a trained convolutional neuronal network with subsequent T2-SI registration slice-by-slice. Received T2-SI curves were subdivided for each cervical level from C2 to C7. Additionally, all levels were subjectively classified concerning a present T2 hyperintensity. For T2-positive levels, corresponding T2-SI curves were compared to curves of age-matched volunteers at the identical level.

**Results:**

Forty-nine patients showed subjective T2 hyperintensities at any level. The corresponding T2-SI curves showed higher signal variabilities reflected by standard deviation (18.51 vs. 7.47 a.u.; *p* < 0.001) and range (56.09 vs. 24.34 a.u.; *p* < 0.001) compared to matched controls. Percentage of the range from the mean absolute T2-SI per cervical level, introduced as “T2 myelopathy index” (T2-MI), was correspondingly significantly higher in T2-positive segments (23.99% vs. 10.85%; *p* < 0.001). ROC analysis indicated excellent differentiation for all three parameters (AUC 0.865–0.920).

**Conclusion:**

This fully automated T2-SI quantification of the spinal cord revealed significantly increased signal variability for DCM patients compared to healthy volunteers. This innovative procedure and the applied parameters showed sufficient diagnostic accuracy, potentially diagnosing radiological DCM more objective to optimize treatment recommendation.

**Trial registration:**

DRKS00012962 (17.01.2018) and DRKS00017351 (28.05.2019)

## Introduction

The radiological assessment in degenerative cervical myelopathy (DCM) is still challenging and requires further improvement due to restricted correlation of imaging alterations and clinical severity of affected patients as well as controversial prognostic utilization [1]. Magnetic resonance imaging (MRI) is the modality of choice, because of the highest tissue resolution and increasing advanced measurements to characterize spinal cord function [1, 2]. Conventional T1- and T2-weighted sequences are routinely applied for the characterization of the anatomical configuration of the spinal canal and the compression of the neuronal tissue as well as for pathological intramedullary signal alterations [1]. Hyperintensities within the spinal cord on T2-weighted images and hypointensities on T1-weighted images are associated with obvious clinical myelopathy [3–5]. Their occurrence is of prognostic relevance and predominantly associated with poor clinical outcome with or without surgical therapy, especially in sharp delineated (“snake-eye”) or multilevel T2 signal increase and when T2 and T1 myelopathy signs occur combined [4–10]. Only slightly, blurry delineated, or postoperatively disappearing T2 hyperintensities are postulated to have better clinical outcomes than the mentioned intramedullary signal aberrations above [11–14]. The character of the intramedullary signal change seems to reflect the progressive tissue destruction in DCM, starting with an intramedullary edema, local demyelination, and ending up with necrosis and spinal cord atrophy [1, 14, 15]. In contrast, there are reports within the literature stating no or inconsistent prognostic reliability for T2 signal alterations [14, 16–18]. Comparable diagnostic performance is also reported for T2*-weighted sequences, whereas they are not part of commonly used clinical spine imaging protocols yet [19]. In summary, the evaluation of conventional MRI myelopathy signs is of importance for determining the diagnosis in suspected DCM, whereas the type and severity seems to be relevant, and the precise outcome prediction is controversial [12, 20].

In clinical routine, the evaluation of such intramedullary signal changes is done subjectively, predominantly resulting in a binary categorization for T1 hypointensities [1]. As mentioned before, there are different types of signal changes on T2-weighted images, whereas a validation or international standardized scoring system is lacking. Additionally, there is consensus concerning the rater-dependency of such subjective classifications. For T2 hyperintensities, prior evaluations showed statistically at least substantial reliabilities for multiple observers with kappa values of 0.60 to 0.82 for binary classifications and intra-class correlation coefficients of 0.75 to 0.87 for ordinal scores [4, 12, 21–23]. However, because treatment decisions are based on those classifications, the relevant amount of incongruent ratings is of clinical importance. There are only few objective evaluations of the spinal cord signal intensity, usually measured through manually placed regions of interests [14, 17, 24, 25]. These evaluations predominantly confirm an inferior prognosis for increased T2 signaling in DCM patients.

Therefore, a more precise and completely objective graduation of intramedullary signal alterations seems to be purposeful for an optimal treatment planning in affected patients. We investigated a fully automated quantification of the T2 signal intensity (T2-SI) of the entire cervical spinal cord based on a high-resolution T2 MRI segmentation.

## Methods

### Study design

This is a matched-cohort analysis of DCM patients and healthy volunteers out of two separate trials, who prospectively underwent identical cervical MRI protocols between July 2018 and Jan 2022. Patients were included through the local outpatient clinic if showing a cervical spinal canal compromise with at least radiological contact of degenerative tissue to the spinal cord in combination with symptoms of degenerative cervical spine disease. Healthy volunteers were recruited through a clinical announcement. Details on inclusion and exclusion criteria have been already published [26]. Both trials were approved by the institutional ethics committee (reference 261/17 and 338/17) and registered at the National Clinical Trials Registry (DRKS00012962 and DRKS00017351). A signed informed written consent was obtained from each participant prior to inclusion.

### MRI acquisition and post-processing

The whole cervical spine was depicted using a common clinically applied T2-weighted 3D sequence acquired on a 3 Tesla scanner (SIEMENS Magnetom Prisma) with a 64-channel head-neck coil (T2 SPACE, voxel size 0.6 mm × 0.6 mm × 1.0 mm, TR 1500 ms, TE 134 ms, Flip angle 105°, GRAPPA PAT: 3, acquisition time 3:53 min).

**Fig. 1:**
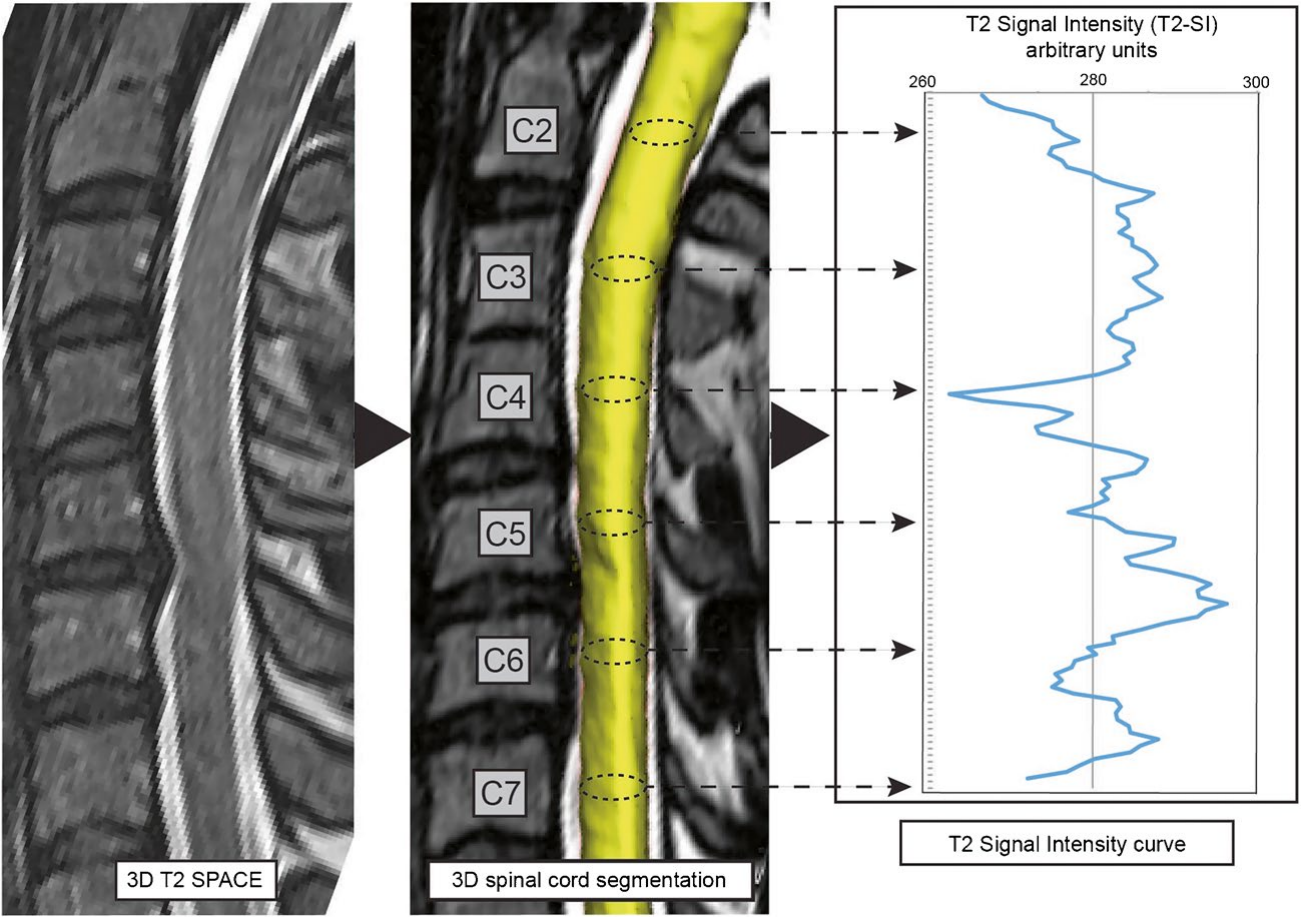
Overview of the post-processing procedure with 3D spinal cord segmentation and generation on the T2-SI curve from C2 to C7 slice-by-slice for a patient with spinal stenosis at the level C5/6 without intramedullary T2 hyperintensities

The next step was a fully automated segmentation of the spinal cord from C2 to C7 based on these 3D T2-weighted images using a trained deep convolutional neural network (CNN). The applied approach involved using nested patches of a fixed matrix size that decrease in physical size. An U-net type architecture was utilized in each scale, with the U-net matrix size set at 32 × 32 × 32 voxels for all scales. A scale pyramid with a depth of four was employed. The architecture of the U-net used was similar to the default U-net configuration presented in the literature [27]. The network was trained with the Adam optimizer with a rate of 0.001 [28]. No systematic tuning was performed, and all labels were trained using binary cross-entropy per channel. The applied algorithm can be accessed online (https://bitbucket.org/reisert/patchwork/wiki/Home). The analyses have been implemented in a special internal software (NORA framework, www.nora-imaging.org). Ground truth segmentations were manually delineated for 15 cases by two examiners, splitting into ten training and five validation cases. Each of the two examiners provides more than 10 years of experience in spinal MRI evaluation. The segmentation reached a Dice coefficient of 0.91. All segmentations were checked for apparent errors by two examiners independently. Additionally, an automated annotation of the vertebral bodies C2 to C7 was trained by another patchwork CNN as anatomical reference. The cross-sectional areas (CSA, mm^2^) of the spinal cord were computed, and the T2-SI was averaged for each CSA slice-by-slice and depicted as T2-SI curve in arbitrary units (a.u.). The spinal cord segmentation and associated T2-SI curve is exemplary shown in Fig. 1.

To compare the particular cervical segments, the mean, standard deviation (SD), and range of the T2-SI of all slices were calculated from the center of the upper to the center of the lower cervical body, respectively.

Representing the extent of T2-SI variability, we introduced the “T2 myelopathy index” (T2-MI) as a new parameter. It was defined as percentage of the T2-SI range in relation to the mean absolute T2-SI within every single cervical segment:

$$\mathrm T2\;\mathrm{myelopathy}\;\mathrm{index}\;\left(\mathrm T2-\mathrm{MI}\right)=\frac{\mathrm T2-\mathrm{SI}\;\mathrm{range}\times100}{\mathrm T2-\mathrm{SI}\;\mathrm{mean}}$$

The complete post-processing including segmentation, T2-SI curve generation, and calculation of the evaluated parameters takes about 3 min.

### Subjective categorization of T2 hyperintensities

For definition of the ground truth, the presence of a T2 hyperintensity within the spinal cord was subjectively evaluated in each cervical level from C2 to C7, receiving five ratings per MRI scan (level C2/3, C3/4, C4/5, C5/6, and C6/7). Accordingly, all segments were classified in a binary fashion as “T2-positive” if a hyperintensity occurred or as “T2-negative” if it did not. To raise reliability, the categorization was done by three independent, blinded, experienced observers. Each of the three observers provides at least 10 years of experience in spinal MRI evaluation. The agreement of all three observers was evaluated through Fleiss’ kappa statistics [29]. For further group comparisons, consensus was defined by majority out of the three ratings when observers disagreed.

### Statistical analysis

For all segments that were subjectively categorized as T2-positive, we generated a control group with age- and level-matched segments out of the healthy volunteers (T2-negative).

For group comparison, Mann-Whitney *U* test for unpaired samples was applied. Gender distribution between both groups was checked through exact binomial test. Overall, *p*-values < 0.05 were assumed to be statistical significant. Normal distribution was evaluated by Shapiro-Wilk-test. In case of non-normal distributed values, median and interquartile range (IQR) were stated. The diagnostic accuracy for the established parameters was defined by ROC analysis. An area under the curve (AUC) of 0.8–0.9 was considered as “excellent” and of > 0.9 as “outstanding” [30]. We additionally applied Youden’s index and the point “closest to the top left” for gaining the most appropriate cut-off-value of the parameters to differentiate for both groups [31, 32]. For statistical calculations, we used Microsoft Excel 16.0 and IBM SPSS Statistics XXVII.

## Results

### Baseline characteristics

Overall, 202 MRI datasets were included for analysis, of which 114 (56.4%) were symptomatic patients and 88 (43.6%) asymptomatic volunteers. Median age of all included participants was 61.0 (IQR 15.2) years and 94 (46.5%) were female.

### Subjective categorization of T2 hyperintensities

Every cervical segment was categorized subjectively by each of the three observers, resulting in 1010 ratings per observer. Inter-rater reliability analysis revealed an absolute agreement of all three observers concerning the T2 hyperintensity categorization in 963 segments (95.3%). Fleiss-Kappa was 0.674 (*p* < 0.001), representing a “substantial” agreement [29].

After forced consensus between the raters, 49 segments were classified as T2-positive, expectedly all out of the patients group. Therefore, 43.0% of the 114 included patients showed a T2 hyperintensity at any level. The predominantly affected levels were C4/5 (*n* = 15) and C5/6 (*n* = 22). There were six T2-positive patients for each level C3/4 and C6/7, and none showing a T2 hyperintensity in C2/3. None of the healthy volunteers showed a T2 hyperintensity.

Each of the 49 T2-positive cervical segments was assigned an age- and level-matched segment out of the healthy volunteers. Accordingly, gender and age distribution between both groups showed no significant differences (Table 1).

**Table 1:** Baseline characteristics of the matched cohorts. *IQR* inter-quartile-range

	T2-positive	T2-negative	*p*-value
Number	49	49	–
Female/male	17:32	25:24	0.189
Age, years, median (IQR)	61.0 (15.0)	61.0 (16.0)	0.929

### Automated T2 signal intensity (T2-SI) analysis

**Fig. 2:**
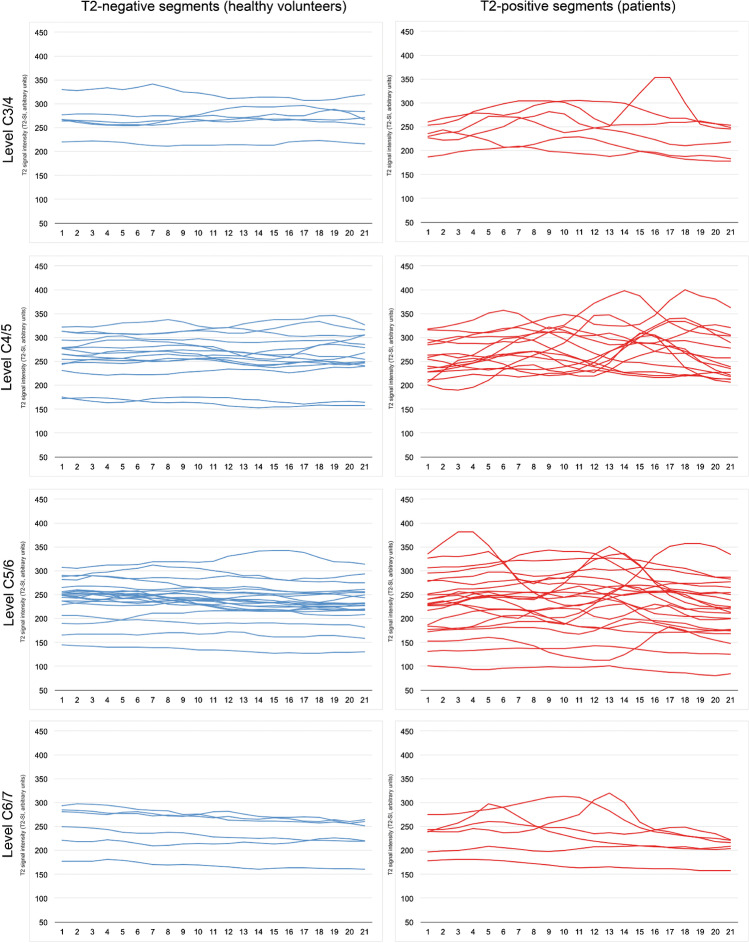
T2-SI curves of all evaluated cervical segments subdivided for the particular cervical levels C3/4 to C6/7. Level C2/3 was excluded due to no patient showing a T2 hyperintensity at this level. The T2-negative segments (blue) of the matched healthy volunteers show apparently more linear curves than the T2-positive segments of affected patients, reflecting higher signal variability

The absolute T2-SI values revealed no significant differences between the T2-positive and T2-negative group (Table 2). The T2-SI curves of all analyzed segments are provided in Fig. 2, divided for both cohorts and the affected cervical level. The curves of T2-positive segments (right) showed a higher variability compared to the matched healthy segments (left). This could be objectivized by a significantly higher T2-SI standard deviation and range within the spinal cord (standard deviation 18.51 (IQR 16.16) a.u. vs. 7.47 (IQR 5.81) a.u., *p* < 0.001; range 56.09 (IQR 52.84) a.u. vs. 24.34 (IQR 17.12) a.u., *p* < 0.001; Table 2). Separate analysis for each cervical level reassured similar differences, however without reaching statistically significance at level C6/7 (Table 2).

**Table 2:** Results for the comparison of T2-positive categorized patients and matched T2-negative segments of healthy volunteers. The absolute T2-SI, T2-SI standard deviation, and range are stated as arbitrary units (a.u.). Values are given as median (IQR). Group comparison was done through Mann-Whitney *U* test for unpaired samples. *P*-values < 0.05 were assumed to be statistically significant and marked as bold type

	All			C3/4			C4/5			C5/6			C6/7		
	T2-negative	T2-positive	*p*	T2-negative	T2-positive	*p*	T2-negative	T2-positive	*p*	T2-negative	T2-positive	*p*	T2-negative	T2-positive	*p*
Number	49	49		6	6		15	15		22	22		6	6	
Absolute T2-SI	250.66 (42.92)	246.67 (60.41)	0.840	266.47 (40.67)	253.06 (67.66)	0.394	263.36 (46.46)	263.50 (66.09)	0.775	237.36 (29.41)	235.35 (88.99)	0.869	251.08 (67.81)	238.83 (61.72)	0.589
Standard deviation T2-SI	7.47 (5.81)	18.51 (16.16)	**< 0.001**	6.68 (6.43)	20.01 (10.41)	**0.015**	7.00 (5.14)	21.35 (12.01)	**< 0.001**	7.44 (8.12)	14.00 (11.76)	**0.001**	7.79 (4.98)	18.55 (22.53)	0.240
Range T2-SI	24.34 (17.12)	56.09 (52.84)	**< 0.001**	19.47 (23.34)	58.66 (37.19)	**0.009**	21.25 (14.04)	71.44 (37.18)	**< 0.001**	25.46 (19.71)	44.45 (38.68)	**< 0.001**	27.68 (14.35)	65.07 (75.06)	0.310
T2-MI	10.85% (7.14)	23.99% (15.21)	**< 0.001**	7.08% (6.37)	23.14% (18.66)	**0.002**	8.41% (6.83)	26.70% (10.35)	**< 0.001**	11.44% (8.57)	19.44% (15.26)	**< 0.001**	11.59% (5.01)	24.81% (28.18)	0.093

**Fig. 3:**
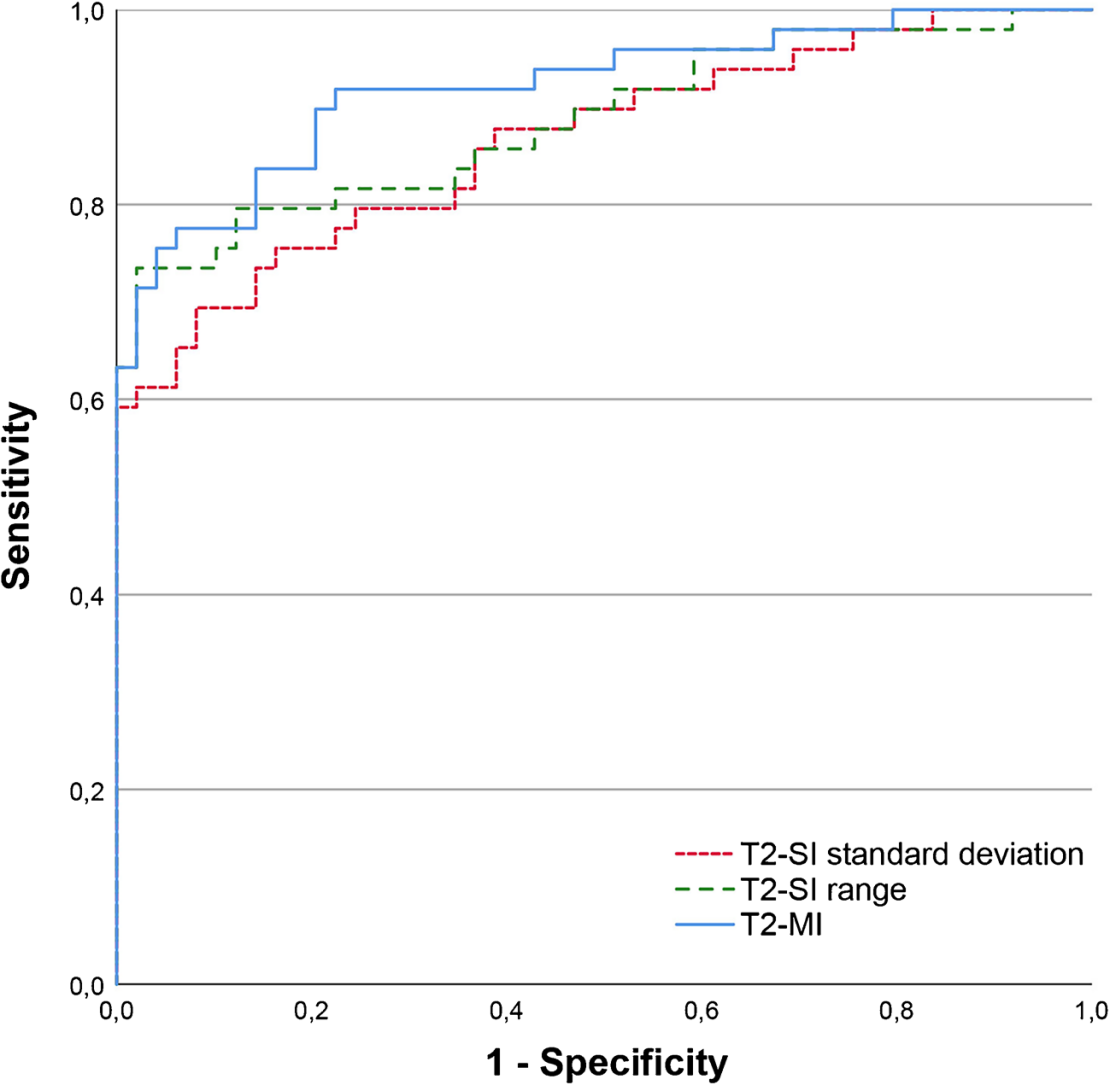
ROC curves for T2-SI standard deviation, range, and T2-MI for separating T2-positive from T2-negative cervical segments. The diagnostic accuracy was throughout “excellent” to “outstanding” (AUC 0.865–0.920).

ROC analysis for the standard deviation and range of the T2-SI showed an excellent discrimination between T2-positive and T2-negative segments (AUC 0.865 and 0.886, Fig. 3). Applying the Youden’s index received an optimal cut-off separating both groups for the T2-SI standard deviation at 13.69 a.u. (sensitivity 69.4%, specificity 91.8%) and for the T2-SI range at 42.31 a.u. (sensitivity 73,5%, specificity 98.0%). “Closest top left values” were 12.20 a.u. (sensitivity 75.5%, specificity 83.7%) and 37.38 a.u. (sensitivity 79.6%, specificity 87.8%), respectively.

### T2 myelopathy index (T2-MI)

**Fig. 4:**
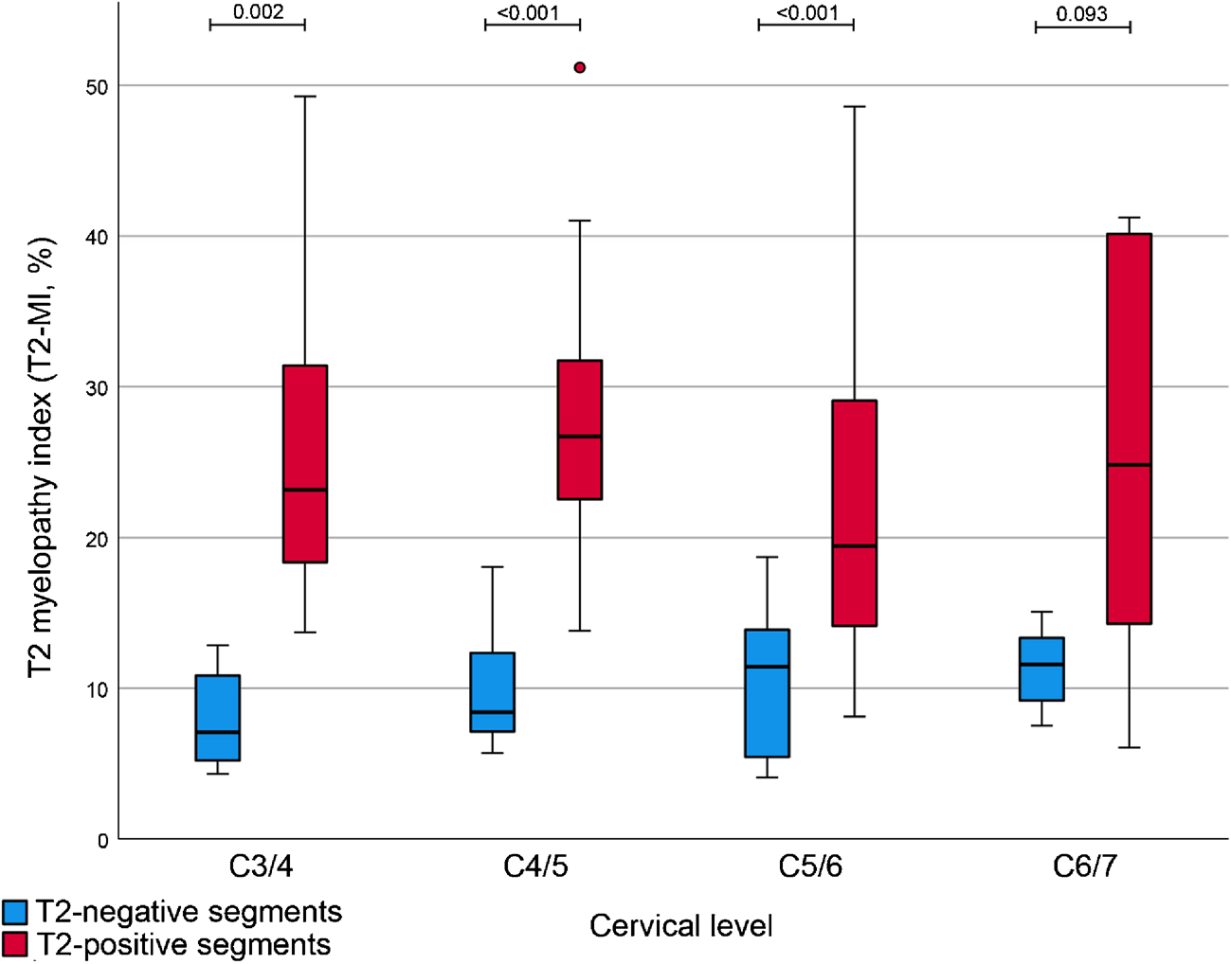
Boxplots comparing the “T2 myelopathy index” (T2-MI) between the T2-negative (blue) and T2-positive (red) group showing significant differences for the levels C3/4, C4/5, and C5/6. There was only a statistical trend for the level C6/7 (*p* = 0.093), despite clearly differing values

The percentage of the T2-SI range in relation to the mean absolute T2-SI per segment, termed as T2 myelopathy index (T2-MI), was correspondingly significantly different between both groups (T2-positive segments 23.99% (IQR 15.21) vs. T2-negative segments 10.85% (IQR 7.14); *p* < 0.001). For the particular cervical levels C3/4, C4/5, and C5/6, this significant difference was reproducible (*p* < 0.001–0.002), whereas level C6/7 showed only a trend (*p* = 0.093, Table 2). Boxplots for the T2-MI separated for the different cervical levels are demonstrated within Fig. 4. For discrimination between T2-positive and T2-negative segments using the T2-MI, ROC analysis showed an outstanding result (AUC of 0.920, Fig. 3). Youden’s index revealed optimal cut-off values for separating both groups at 15.32% (sensitivity 77.6%, specificity 93.9%) and 17.60% (sensitivity 75.5%, specificity 95.9%). The “closest top left value” of the ROC curve was at 14.14% (sensitivity 83.7%, specificity 85.7%).

## Discussion

We present a new quantification procedure for the T2-SI of the spinal cord that was able to differentiate between cervical segments of DCM patients with a present T2 hyperintensity and healthy volunteers based on the variability of the signal intensity. This fully automated process has the potential for diagnosing radiological DCM objectively and standardized, thus optimizing the recommendation for treatment. The received sufficient diagnostic accuracy of the established parameters and their cut-off values have to be confirmed through further investigations and correlated to clinical symptoms of affected patients.

This T2-SI quantification tool is based on a fully automated segmentation procedure of the spinal cord, based on a trained deep CNN showing sufficient accuracy. Minor interferences of compromising degenerative tissue and spinal cord in high-grade stenosis are conceivable due to nearly iso-intense signaling at those borders. However, two independent observers did not detect any apparent segmentation error. The next step was the generation of a T2-SI curve for the whole cervical spinal cord, averaging the T2-SI within every slice (Fig. 1). The advantage is an objective and highly resoluted analysis of the T2-SI and its variability from slice to slice with a correspondingly registration of already minor changes of the signaling. To our knowledge, such an automated signal intensity analysis of the spinal cord is implemented in a routinely fashion for the first time, and each cervical level can be consecutively screened for abnormal signal alterations. Several semi- to fully automated spinal cord segmentation procedures are reported within the literature [33, 34]. A large elaborated study by Gros et al. on 1.042 imaging datasets of predominantly multiple sclerosis patients reported sufficient segmentation reliabilities with a Dice coefficient of 0.88–0.95 using a related CNN, which is therefore comparable to our results [19].

The exact position of the T2 signal alteration within the spinal cord and the type of their boundaries was not addressed in our evaluation due to averaging of every cross-section. However, the sharp or blurry demarcation of T2 hyperintense areas was a relevant prognostic factor in previous subjective classifications, which has to be considered as limitation [4–14]. A reported CNN-based detection of multiple sclerosis lesions and their shape showed sufficient accuracies, which seems to be an option for the detection of myelopathic lesions similarly [19]. Two MRI examinations showed a prominent central canal, which could affect the signal intensity evaluation. For the required 3D segmentation of the spinal cord, a slice thickness of maximum 1 mm is necessary and almost possible in an adequate acquisition time due to enhanced MRI scanner technology. Our whole evaluation procedure takes about 7 min, consisting of nearly 4-min acquisition time and about 3 min post-processing, which seems to be implementable in every clinical “all-day” setting. The applied 3D T2 SPACE sequence has the disadvantage of a worse signal-to-noise-ratio and image contrast compared to conventional 2D T2-weighted sequences, usually used for subjective categorization of T2 hyperintensities [35]. However, the diagnostic quality is acceptable for the evaluation of intramedullary T2-SI variations, demonstrated through our results.

The absolute T2-SI values are not interindividually comparable due to known specific imaging conditions for each MRI scan [14]. That could be confirmed by these results, showing no significant differences between both groups for the absolute mean values (Table 2). Besides the T2-SI variability represented by the standard deviation and range, we introduced the new, inter-individually comparable parameter T2 myelopathy index (T2-MI). The T2-MI represents a conventional percentage representing the extent of T2-SI heterogeneity in a metric scale, increasing with a higher signal variability within every evaluated segment. This parameter could also reliably distinguish between subjective T2-positive and inconspicuous cervical levels with an outstanding AUC of 0.920 and optimal diagnostic accuracy using a cut-off of approximately 15%, depending on the applied statistical test (Fig. 4). Separate analysis for every particular cervical level from C2 to C7 revealed corresponding significant differences, whereas level C6/7 could only show a statistical trend (*p* = 0.093). Nevertheless, even at level C6/7, both groups showed clearly distant values for the T2-MI of 11.6% (T2-negative) versus 24.8% (T2-positive).

**Fig. 5:**
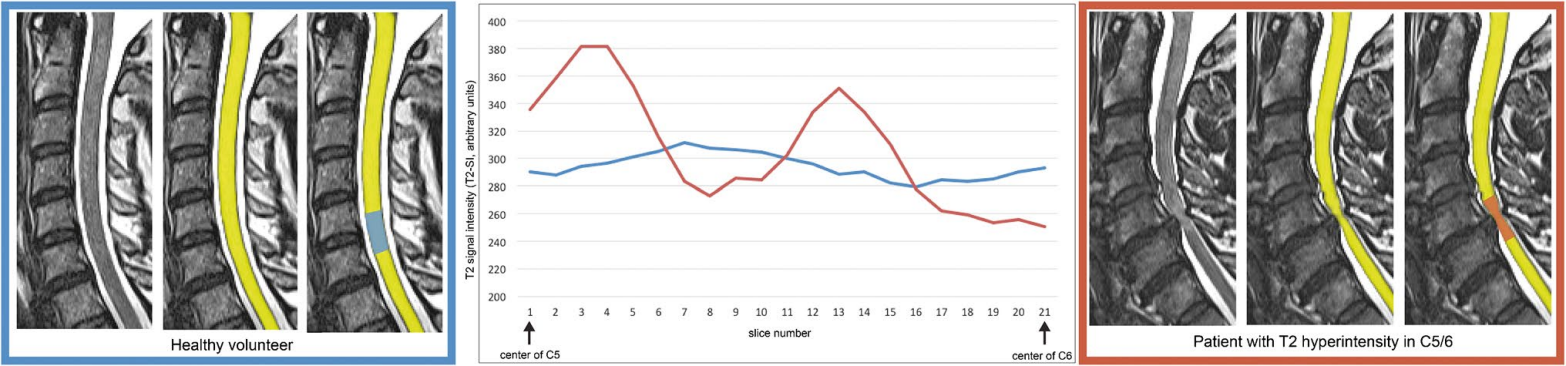
Example of the spinal cord segmentation procedure for cervical level C5/6 and the corresponding T2 signal intensity (T2-SI) curves of two representative subjects for both groups: The signal intensity curve for a T2-positive patient (red) shows a higher range and therefore variability of the T2-SI through the affected segment compared to a matched T2-negative healthy volunteer (blue)

Figure 5 depicts two exemplary segments at level C5/6 with a typical T2-SI curve for a patient showing a high signal variability (red curve) and a healthy volunteer with a nearly linear curve (blue). The extent of the variability might reflect the dimension of intramedullary alterations and is expected to be associated to the severity of myelopathy symptoms and anticipated outcome after treatment. All three parameters reflecting the T2-SI variability have the advantage of grading alterations within the spinal cord in a more dedicated, metric setting and not dealing with binary or ordinal subjective scales. Therefore, a better correlation to the clinical affection and different stages of DCM is expected. A limitation of this data is that we show isolated radiological parameters without linking it to clinical affection. We expect that associated to mentioned prior evaluations, the extent of the signal variability correlates with clinical impairment, which is already subject of further clinically centered investigations. Independently of the clinical correlation, this evaluation procedure works fully automated without dealing with rater-dependency, and it is applicable for every high-resoluted T2-weighted sequence in advance, which gains a new step in radiological assessment of DCM.

To summarize the main limitations of our study, we predominantly have to mention the still limited patient population as basis for our analysis. Additionally, it has to be taken into account that the applied parameters standard deviation, range, and T2-MI are arbitrary. The extent, position, and the boundary type of the T2 signal alteration within the spinal cord was not addressed in this evaluation, which, however, should be relevant for the characterization of DCM patients.

For the future, diagnostic accuracy and prognostic information for affected patients should be improved by combining different MRI modalities. Especially diffusion-based techniques, such as diffusion microstructural imaging (DMI) or diffusion tensor imaging (DTI), may allow conclusions about alterations of the axonal integrity and intramedullary free water distribution [18, 26, 36–38]. Cerebro-spinal fluid and spinal cord motion, assessed by phase-contrast imaging, or even specific metabolite configurations from magnetic resonance spectroscopy may provide additional pathodynamic information [39–41]. We are working on a multimodal MRI assessment integrating different modalities and correlate the imaging information to the symptomatic and electrophysiology to optimize clinical treatment decisions and outcomes for DCM patients.

This evaluation represents one step for this approach with implementation of a routinely applicable, fast and observer-independent assessment of intramedullary T2 signal alterations and transferring this into a quantifiable setting.

## Conclusions

This fully automated T2 signal quantification of the spinal cord was able to reveal a significantly different variability of the T2-SI in patients with DCM and present T2 hyperintensities compared to healthy volunteers. Whilst the absolute T2-SI values are not applicable due to known individual imaging conditions, we introduced the new, inter-individually comparable T2 myelopathy index (T2-MI), showing corresponding significances to the extent of signal variability represented by the standard deviation and range of T2-SI at every cervical level.

This automated, innovative procedure has the potential for diagnosing radiological DCM more objectively and standardized, thus optimizing the recommendation for treatment. The determination of more detailed cut-off values associated to the different stages of DCM and its diagnostic accuracy is under further investigation.

## Data Availability

Used data can be provided on reasonable request.

## Abbreviations

*a.u*.: arbitrary units
*AUC*: area under the curve
*CNN*: convolutional neural network
*CSA*: cross-sectional area
*DCM*: degenerative cervical myelopathy
*DTI*: diffusion tensor imaging
*MRI*: magnetic resonance imaging
*T2-MI*: T2 myelopathy index
*T2-SI*: T2 signal intensity
